# Dynamics of functional connectivity at high spatial resolution reveal long-range interactions and fine-scale organization

**DOI:** 10.1038/s41598-017-12993-1

**Published:** 2017-10-06

**Authors:** Maria Giulia Preti, Dimitri Van De Ville

**Affiliations:** 1 Institute of Bioengineering, Center for Neuroprosthetics, Ecole Polytechnique Fédérale de Lausanne (EPFL), Lausanne, Switzerland; 2 Department of Radiology and Medical Informatics, University of Geneva, Geneva, Switzerland

## Abstract

Dynamic functional connectivity (dFC) derived from resting-state functional magnetic resonance imaging sheds light onto moment-to-moment reconfigurations of large-scale functional brain networks. Due to computational limits, connectivity is typically computed using pre-defined atlases, a non-trivial choice that might influence results. Here, we leverage new computational methods to retrieve dFC at the voxel level in terms of dominant patterns of fluctuations, and demonstrate that this new representation is informative to derive meaningful brain parcellations, capturing both long-range interactions and fine-scale local organization. Specifically, voxelwise dFC dominant patterns were captured through eigenvector centrality followed by clustering across time/subjects to yield most representative dominant patterns (RDPs). Voxel-wise labeling according to positive/negative contributions to RDPs, led to 37 unique labels identifying strikingly symmetric dFC long-range patterns. These included 449 contiguous regions, defining a fine-scale parcellation consistent with known cortical/subcortical subdivisions. Our contribution provides an alternative to obtain a whole-brain parcellation that is for the first time driven by voxel-level dFC and bridges the gap between voxel-based approaches and graph theoretical analysis.

## Introduction

Spontaneous fluctuations of brain activity as measured by resting-state functional magnetic resonance imaging (fMRI) have shown to be organized in terms of large-scale functional networks^1^. One powerful tool to investigate brain networks is to build graph representations where nodes are associated to brain regions, and edge weights are given by functional connectivity (FC) measured as Pearson correlation between the regional averaged timecourses^2,3^. Functional connectomes have revealed system-level aspects related to scale-free^4^, small-world^5^, modular^6^, and rich-club organization^7^. In this framework, the choice of a brain parcellation is essential, as the full spatial size of the data is prohibitive in terms of computations and storage; i.e., dealing with all gray-matter voxels and building a voxel-by-voxel functional connectome would easily reach the order of 10^10^ entries. The construction of parcellations or atlases is based on homogeneity assumptions that can be anatomical or functional, leading to 100–1000 regions and thus reducing the number of connections to the order of 10^4^–10^6^. Well-known anatomical/cytoarchitectonic parcellations are based on manual segmentation of a single subject, such as the Brodmann atlas^8^ and the automated anatomical labeling (AAL)^9^, or on a population average^10–13^. Whole-brain parcellations based on the functional connectivity of the cortical compartments^14^ have been proposed based on clustering^15–25^, which can be spatially constrained^26,27^, or informed by independent component analysis^28–30^. Some studies also combined different types of anatomical and functional information, to further increase the accuracy of the parcellation^31,32^. The granularity is a compromise between the spatial variability of the functional signals and anatomical interpretability^22,25,33,34^. Different parcellation schemes can lead to substantially different network structures and statistics, and thus have an important influence on the analysis^25,35–37^. In addition, there is no gold standard or optimal solution, as it might depend on the data quality and resolution. However, due to computational limitations, only a limited number of studies has explored graph representations at the voxel level with direct computation of the connectivity matrix^4,38–44^. Others^45–52^ investigated eigenvector centrality of functional networks with a fast computation method introduced by^53^. In these studies, a fine-grained analysis of resting-state cortical hubs revealed differences with the known resting-state networks^38^, allowed to link for the first time connectome modifications to specific genetic variants^52^, as well as highlighted new properties of functional network topology^4,40^, and demonstrated disease-related changes to functional network properties^38,39,43,46,49–51^. Recent research has focussed on retrieving the moment-to-moment fluctuations in FC that remain hidden to measures computed over a whole resting-state scan of several minutes^54–57^. The most commonly applied “dynamic” FC (dFC) measures are derived from windowed correlation values between the signals of different brain regions. In such a setting, the number of connectomes obtained for a single run can easily reach 100–1000, which then requires additional steps such as clustering^58,59^ or dimensionality reduction^60^ to establish “building blocks” of patterns that occur over time. The abondance of data generated by dFC even further motivates practitioners to rely on (coarse) spatial parcellations. However, to date, it is unclear whether such an approach is sufficient to capture all the richness available in dFC. This points towards the need for techniques that allow bridging the gap between voxel-level approaches and graph-theoretical analysis.

In this work, by providing a technical solution for data-driven analysis of dFC at the voxel level, we investigate to what extent fluctuations in FC are able to drive themselves a meaningful parcellation, and whether they would be informative both on long-range and fine-scale organization. To overcome the challenges related to computational and storage requirements, we built upon the concept of eigenvector centrality^41,53^ to obtain an implicit representation of the voxel-level connectivity matrix by its underlying dominant spatial map. Due to the limited amount of information captured by windowed correlations, this approach yields a good approximation of the voxel-level connectivity matrix. The dominant maps of subsequent windows are then concatenated across participants, followed by consensus clustering^61^, to obtain the most representative dominant patterns (RDPs). We then established for each voxel a label, by assessing its positive or negative contribution to each of the RDPs. We obtained 37 dFC patterns that regroup voxels with the same dynamics in terms of dFC fluctuations. These patterns reflect long-range interactions of known resting-state networks. Splitting each of the dFC patterns in contiguous regions leads to 449 regions in total, which reveal fine-scale organization in terms of meaningful subdivisions.

## Results

### Representative Dominant Patterns of dFC

**Figure 1:**
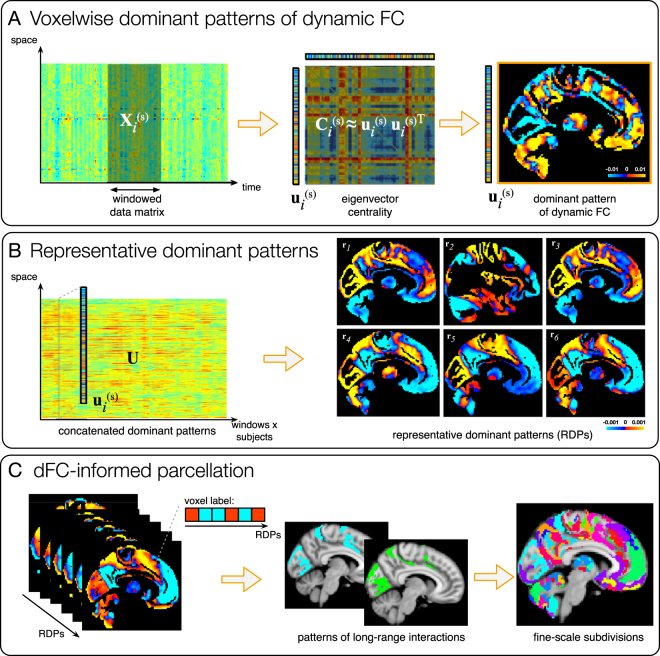
Illustration of the data processing pipeline and results. (**A**) Eigenvector centrality applied with a sliding-window approach allows to extract window-specific voxelwise dominant FC patterns for every subject *s*. (**B**) Dominant patterns are aggregated across time/subjects and *k*-means clustering is applied yielding the most representative dominant patterns (RDPs), characterizing resting-state dFC in the population. (**C**) All possible combinations of sign with which voxels belong to the obtained RDPs are considered and voxels displaying the same combination are aggregated together, generating a parcellation including patterns of long-range interaction. These are subdivided in contiguous regions, yielding a parcellation with fine-scale subdivisions.

The complete data processing pipeline is illustrated in Fig. 1.

**Figure 2:**
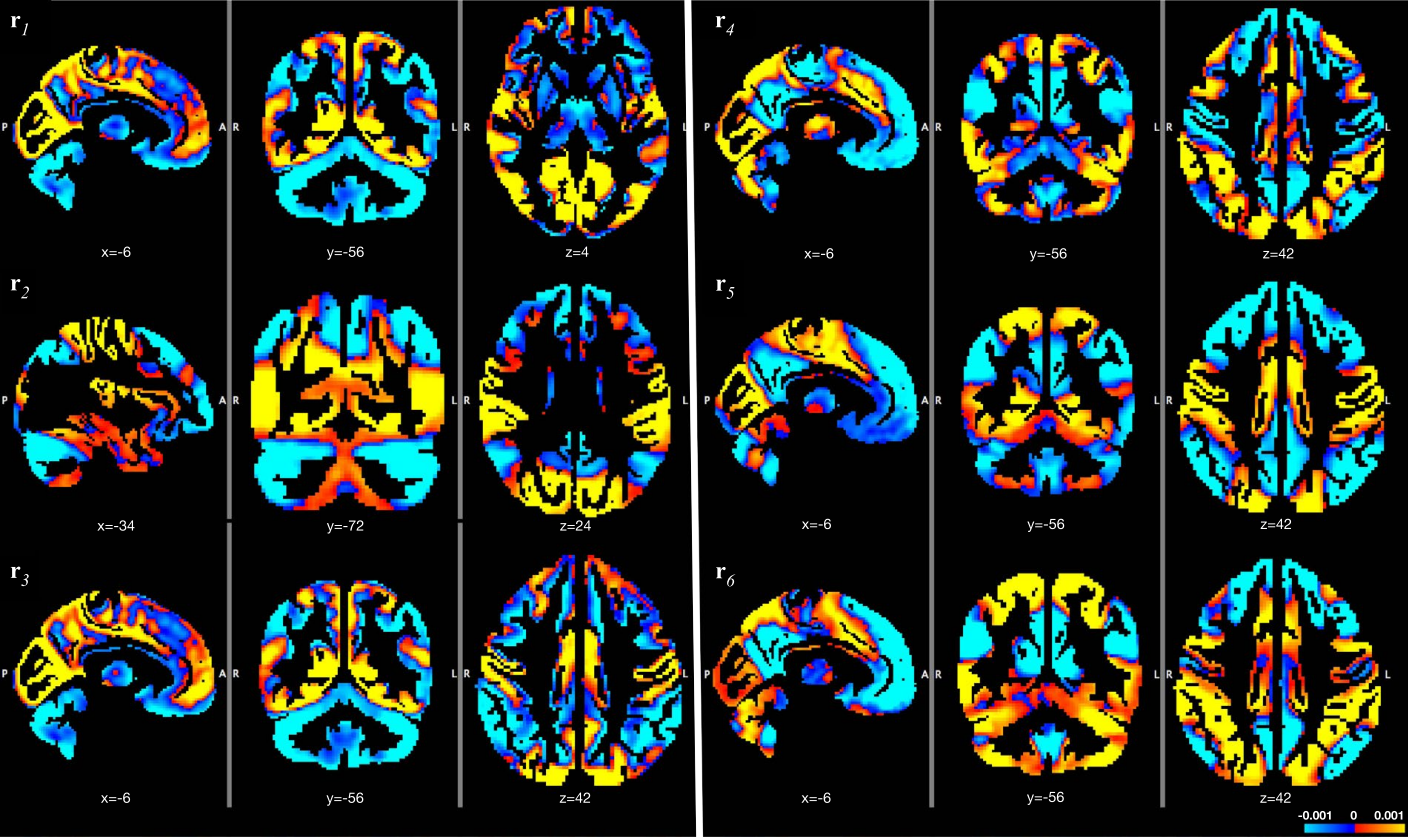
Representative dominant patterns characterize the spatial configurations of dFC fluctuations. They are obtained by *k*-means clustering of the time-varying dominant patterns of dFC over all subjects. MNI coordinates are displayed.

Eigenvector centrality applied to the FC matrices obtained with a sliding-window approach yielded window-specific voxelwise dominant FC patterns for every subject (Fig. 1A). These maps can be interpreted as rank-1 approximations of the connectivity at every time point. *k*-means clustering of the dominant patterns, aggregated across time and subjects, yielded the RDPs showed in Fig. 1B and more in detail in Fig. 2 and Supplementary Figs S1–S6. Ten-fold resampling on the *k*-means clustering suggested *K* = 6 as the most suitable number of clusters. From the out-of-fold clustering error plotted in Supplementary Fig. S7, we observe in fact decreasing trends up to *K* = 6, before the median and spread of the error increases for *K* ≥ 7.

RDP 3, which shows a clear contrast between primary sensory/sensorimotor networks and cerebellum/subcortical/frontoparietal/temporal regions, is the one that temporally occurs most often (28% of total time), followed by the other RDPs, all between 11–17%. In general, the RDPs reflect combinations of full and partial known resting-state networks, including default-mode network (DMN), fronto-parietal network (FPN), salience, sensorimotor, primary sensory, visuospatial networks^29^. Supplementary Fig. S8 quantifies the relative overlap between each RDP and the conventional resting-state networks^29^ and main subcortical regions^9^. Some of the major trends interesting to observe from the RDPs are reported in the following. For instance, in RDP 5, the DMN and the bilateral FPN are opposed against the primary sensory and sensorimotor regions. In RDPs 4 and 6, the DMN is segregated in its ventral and dorsal parts^29^, where the superior precuneus now goes along with regions related to attention (i.e., anterior and posterior salience network, visuospatial network) while the remaining DMN co-fluctuates with the language network. In RDP 3, the precuneus appeared segregated in an anterior/posterior way. A more profound analysis of precuneus’ subdivisions by combining the information from all RDPs will be presented below. Cerebellar and subcortical regions were also retrieved in association/opposition to many different cortical regions, sometimes contributing globally (e.g., in RDPs 1 and 3) or in a segregated way.

### Parcellations Driven by Dynamic FC

**Figure 3:**
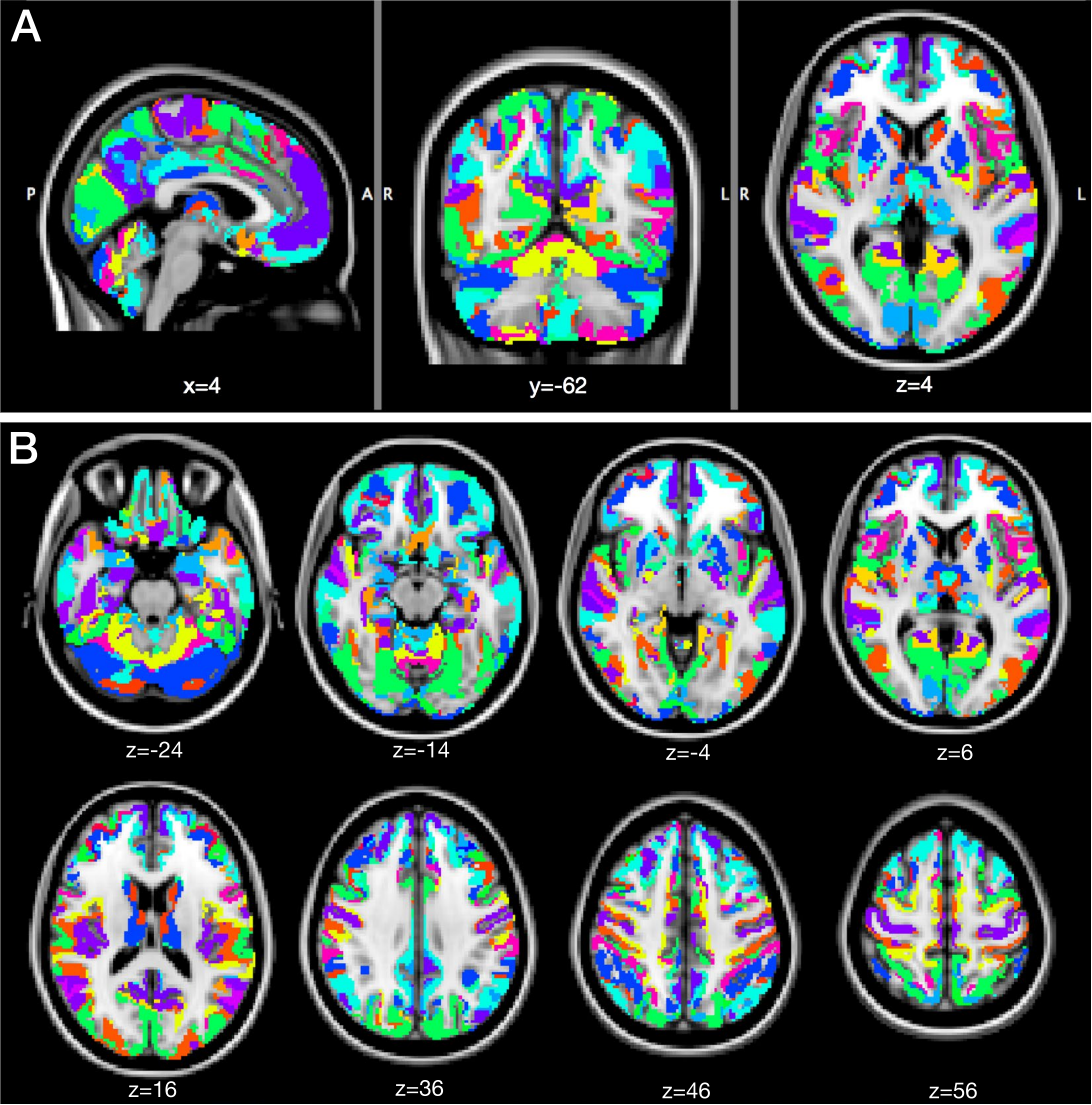
dFC-driven brain parcellation. The 36 unique labels are overlaid onto a brain MNI template, in (**A**) sagittal, coronal and axial views, and (**B**) multiple axial views (MNI coordinates are reported).

Based on their positive/negative contributions to the 6 RDPs, labels were attributed to all voxels (Fig. 1C). The maximum number of labels is 2^6^ = 64, and initially we found 63. However, after pruning the smallest areas, we only retained 37 unique labels. In Fig. 3, we visualize the corresponding parcellation. These patterns are not contiguous and reflect long-range interactions captured by dFC. To quantify the distributed nature of each pattern, we computed the Euclidean distances between all possible pairs of contiguous clusters (i.e., their centroids) within the pattern. These distances ranged from 30 to 90 mm with an average and standard deviation of 71.70 ± 16.86 mm.

The hemispherical symmetry of the patterns was also remarkable, especially for the largest ones. 98% of the covered cortical voxels were assigned to labels whose symmetry index (SI) was between −1 and +1, where 0 is perfect symmetry and ±2 is complete asymmetry. Next, we obtained the final fine-scale parcellation by splitting the patterns in their contiguous regions, which resulted into a total of 449 areas (Fig. 1C). To demonstrate that meaningful subdivisions are recovered, we report more details for specific brain areas in the following.

#### Precuneus

**Figure 4:**
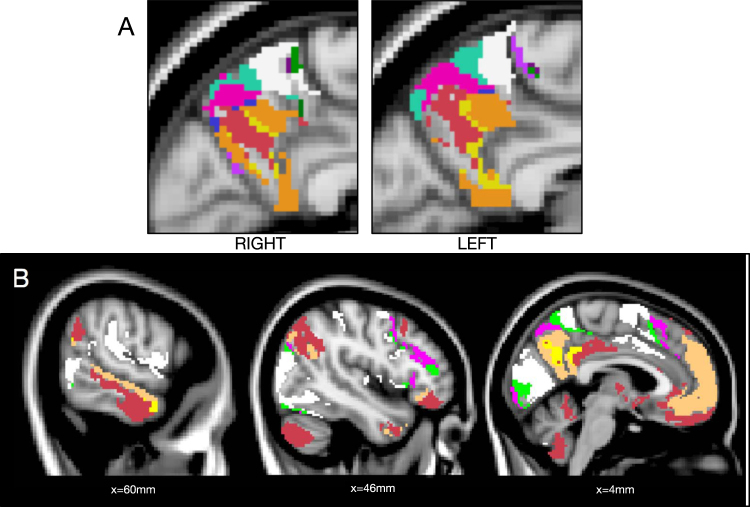
dFC-driven precuneus parcellation. (**A**) The 14 labels of the parcellation that contribute to the precuneus are shown, selectively for this structure (right and left sagittal views). (**B**) The full brain view of the six atlas labels contributing the most to the precuneus are displayed, showing the brain areas which are dynamically connected to this structure (three sagittal views, MNI coordinates are reported). The dorsal precuneus (white, green and pink ROIs) mainly project to anterior salience, SMA, temporal and visual areas, while the ventral precuneus (orange, red and yellow ROIs) was found to be mainly involved in the DMN, FPN and language network.

The bilateral precuneus was segregated in 14 unique labels (including 41 contiguous regions) with remarkable hemispherical symmetry (Fig. 4A).

Consistently with previous reports^62,63^, the dorsal precuneus mainly splits into an anterior, a medial and a posterior part (i.e., white, green and pink areas), while the more ventral precuneus is divided into three other subportions (i.e., orange, red and yellow areas) (see also Supplementary Fig. S9).

We can now look at the long-range interactions of each label (Fig. 4B). For instance, the dorsal-anterior part (white) relates to anterior salience (dorsal anterior cingulate cortex and insula), supplementary motor area (SMA), mid-cingulate cortex, primary visual, visuospatial and temporal (superior and middle) areas. The middle dorsal part (green), similarly, is mainly connected to anterior salience (frontal superior medial and mid-frontal areas) and visual areas, in addition to a small thalamic subportion. The posterior part (pink) is instead connected to SMA and visual cortex. Differently, the ventral precuneus was found to be mainly involved in the DMN (dorsal DMN in orange and posterior DMN in yellow) and combinations of DMN, FPN, and language networks (red).

#### Cerebellum

**Figure 5:**
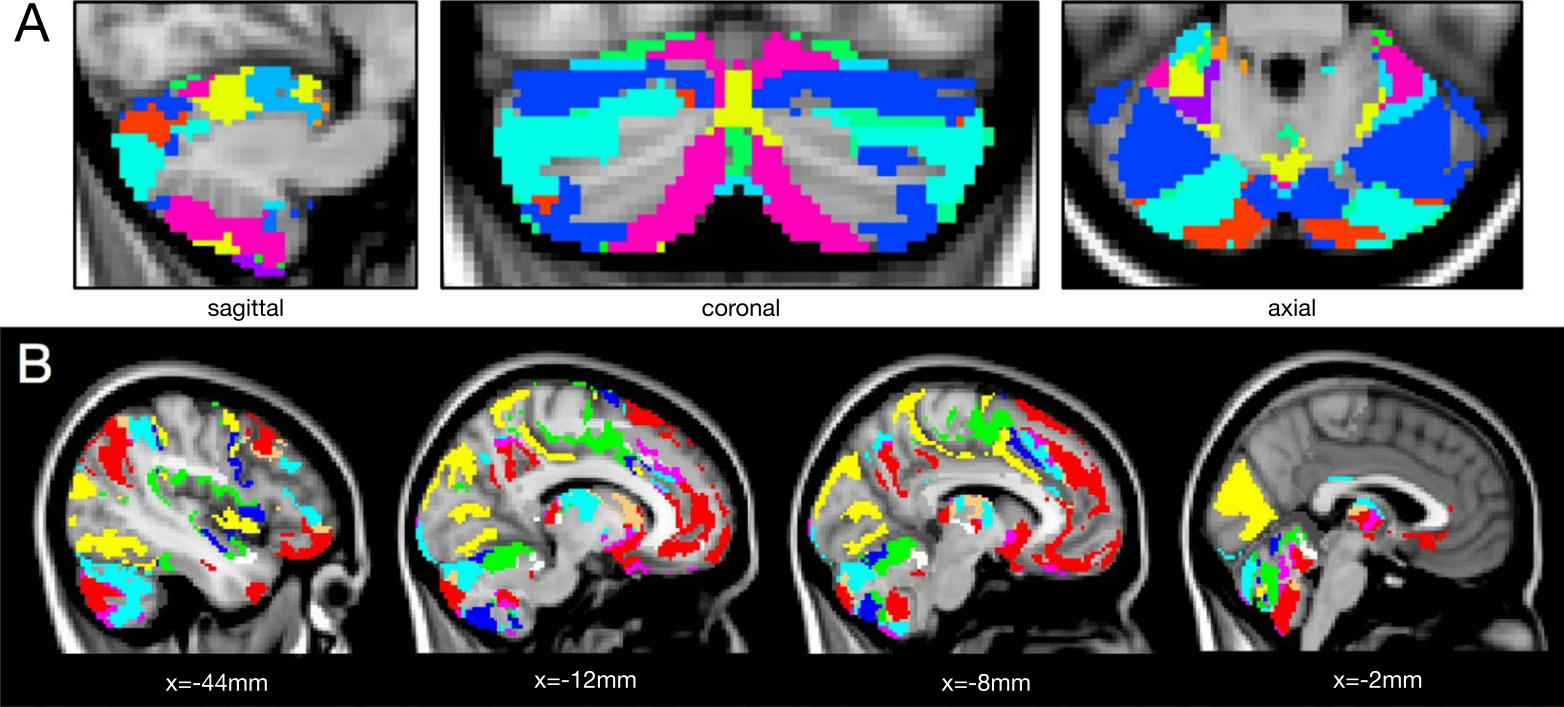
dFC-driven cerebellum parcellation. (**A**) The 17 labels of the parcellation that contribute to the cerebellum are shown, selectively for this structure. (**B**) Connectivity of the cerebellum. The atlas labels which contribute to the cerebellum are shown in different colors (four different sagittal views, MNI coordinates are reported). It is possible to highlight meaningful cortical projections of the segmented cerebellar subregions. In particular, the four largest ROIs in the cerebellum segmentation show here the following connections: ROI 1 (red) includes the intersection between Crus I/II and DMN areas (see middle panels); ROI2 (light blue) includes other Crus I/II portions and fronto-parietal areas (first panel on the left); ROI 3 (green) shows cerebellar lobule VI and sensorimotor cortex (middle panels); ROI4 (blue) displays lobuli VI, VIIb and VIII connecting with anterior/posterior salience network (third panel from the left).

The cerebellum was segregated in 17 unique labels (composed of 60 contiguous regions), again with striking interhemispherical symmetry (Fig. 5A). On one hand, from an anatomical point of view, we found partially but not fully consistent results with existent anatomical atlases (AAL and SUIT^64^); e.g., our labeling marks the boundary between different known anatomical cerebellar subdivisions, such as lobules IV-V, VI, Crus I and II (see Supplementary Fig. S10).

On the other hand, the long-range connectivity of our cerebellar subdivisions showed connections between different cerebellar labels and distinct and meaningful cortical networks. This involved several extended regions of the cortex, including motor, cognitive and association areas. In Fig. 5B, we limit the view on the parcellations by only those labels that occur in the cerebellum. The four ROIs including larger portions of the cerebellum (ROI1 shown in red, ROI2 in light blue, ROI3 in green, and ROI4 in blue) display the following cerebello-cortical connections: ROI1, including the Crus I/II intersection and part of cerebellar lobule IX, selectively connects to regions of the DMN (posterior cingulate cortex (PCC), precuneus and ventromedial prefrontal cortex), while ROI2, including other portions of Crus I/II and lobule 7b, is recruited together with bilateral FPN, besides the anterior and posterior salience networks, inferior temporal and subcortical regions (thalamus, caudate, pallidum, putamen); ROI3 and 4, instead, mainly include portions of the same cerebellar regions (lobuli VI, VIII and IX, as well as IV-V for ROI3), but connects to different areas in the cortex; i.e. the sensorimotor and primary motor networks, middle cingulate and SMA in the former case, the anterior/posterior salience network, SMA and mid-frontal regions in the latter one.

### Reproducibility Analysis

The RDPs were highly reproducible with an independent dataset (using another session). First, the spatial correlation between the RDPs of the two datasets is 0.92. Second, the dFC-driven parcellations are also comparable as characterized by the following similarity metrics: Adjusted Mutual Information (AMI) = 0.63, Rand Index (RI) = 0.96 and Adjusted Rand Index (ARI) = 0.3. The degree of similarity assessed by these indexes revealed to be in line with previous studies where multiple datasets are compared across different acquisitions^15^.

As expected, the analyses with different dFC window lengths led to highlighting different properties of FC dynamics. Increasing the window length to *N*_*T*_ = 90 s reduces in fact by construction the observable dynamics spectrum and, as a consequence, less variable RDPs were obtained. In fact, the cosine inter-cluster similarity between RDPs resulted >0.94 for three pairs cluster centroids, meaning that mainly three of the six original patterns were retrieved. This led to a parcellation with lower number of regions (32 long-range labels and 399 contiguous regions). Shortening the window to *N*_*T*_ = 30 s, instead, increases the observable dynamic range, and in fact leads to higher variability in the retrieved RDPs (the cosine inter-cluster similarity between RDPs was always <0.68) and therefore a higher resolution parcellation (49 long-range labels and 561 fine-scale subregions).

## Discussion

### Methodological Considerations

We provided here a novel approach to obtain a brain parcellation, which is driven by dFC at the voxel level. Building upon efficient numeral implementations to compute leading eigenvectors of large matrices, we showed that it is feasible to obtain dominant patterns of brain activity that provide implicit representations of voxel-wise dFC matrices. The temporal redundancy of these dominant patterns across windows was then exploited using consensus clustering, leading to a limited number of 6 representative dominant patterns. These RDPs were expressed uniformly across subjects. Then, for each voxel, we assigned a label based on its signs in all RDPs. Each of the 37 unique labels identifies a pattern that reveals both long-range interactions and fine-scale organization, leading to 449 contiguous regions in total.

The processing pipeline contains several steps to highlight dynamic changes in connectivity. First, the connections’ timecourses were temporally demeaned before computation of dFC, which effectively subtracts the baseline stationary FC from the fluctuations of dFC. A preliminary analysis^65^ showed that this demeaning performed at the level of connections does not affect the results at the voxel-level in terms of dominant patterns, but we chose here to keep this step in order to build a voxelwise dFC setting consistent with previous atlas-based dFC analysis^60^, as well as to remove any bias introduced by inter-subject variability of stationary FC. Second, global signal regression (GSR) was performed on the fMRI timecourses. Several controversies have been generated in the past by the application of GSR to resting-state fMRI data, with the subsequent observation of negative correlations^66,67^. A recent study from ref.^68^, aimed to reach a consensus concerning this long-debated matter, asserts that whether to apply GSR or not depends on the specific scientific question and must be considered when interpreting results. Repeating the analysis with and without GSR is suggested to help the interpretation. In our case, the same analysis conducted without this regression yielded really similar RDPs, but included also a strong global pattern interfering with the functional network patterns. Therefore, the application of GSR had the only effect of discarding this component, known to be present and reflecting global fluctuations of the connectivity^60^, allowing to better retrieve the networks that dynamically reconfigure on top of it.

The effect of the window length, a crucial parameter for dFC computation^57^, appears also important for the current analysis. In fact, as known, substantially increasing the window length leads to less fluctuations, translating in our case into a poorer set of RDPs and, as a consequence, in a coarser parcellation. On the contrary, shorter window lengths increase the temporal scale at which dFC is measured, translating into more variable RDPs and a finer parcellation. However, shorter window lengths can also become critical for multiple reasons. First, the original fMRI timecourses need to be high-pass filtered to avoid spurious fluctuations due to aliasing^69–71^—such high-pass filtering can potentially eliminate useful information. Second, the window shortening reduces the number of samples considered for the computation of the connectivity inside the window, and therefore the reliability of the correlation measure itself. The choice of a window length of 60 s, sustained by several previous literature studies (see ref.^57^ for a review), seemed to be a reasonable trade-off between these aspects.

It should also be noted that eigenvector centrality is influenced by the voxels’ degrees that consists of the (implicit) sum of Pearson correlations with all other voxels; therefore, there could be an indirect effect of area size, topographic organization, or system size^72^. In this perspective, the temporal clustering applied in this work is an important additional step leading to the RDPs that reveal how FC patterns change over time and it is these temporal fluctuations that drive the subsequent parcellations.

There are several extensions that could be considered for future work. First, even though eigenvector centrality mapping showed to successfully capture neural architecture at the voxelwise scale^41,52,53^, a single dominant pattern per temporal window and the associated rank-1 approximation does not capture all richness of the dFC matrix. We can also interpret this pattern as a kind of windowed co-activation pattern (CAP)^73,74^; i.e., the spatial pattern that best explains the activity during the window length in terms of mean squared error with the empirical data. Considering multiple dominant patterns per temporal window to build a more complete low-rank approximation can be done, but there are open questions on how to perform meaningful clustering of these representations. Second, the current labeling is done according to a hard assignment based on the signs of the RDPs, which could be improved by using soft clustering and support overlapping regions. Further, hierarchical clustering based on Hamming distance between the voxels’ binary labels could be performed to aggregate labels and take into account the overlap between RDPs. This can be particularly useful if a coarser parcellation is desired.

### Neuroscientific Relevance

The dominant patterns extracted from the sliding-window observations are reflecting the main patterns of variation on top of globally averaged behavior. These patterns are contrasting large-scale functional networks that are known to be involved in spontaneous thought—including DMN, FPN, salience, and attention networks—^29^ with, however, several important spatial differences occurring over time to justify the emergence of 6 unique RDPs. The interplay of these networks and their subnetworks could provide a handle on fitting models of dynamics of mind wandering^75^. The recruitment of various DMN subsystems at different instants—for instance, appearing in the RDPs as contraposition between anterior/posterior and dorsal/ventral parts—is in line with the several reports highlighting the functional heterogeneity of the DMN^63,75^. This further underlines a more complex interplay between task-negative and task-positive networks (TPN), the latter being involved in task engagement and goal-directed thought^76^. Recently, these two networks have shown not to be constantly anti-coupled, revealing the presence of brain states which are mixtures of DMN and TPN^63^, as well as in terms of occurrences with simultaneous activation or deactivation^77^. Such a selective recruitment of DMN subsystems might be a potential correlate of moment-to-moment variations in mind-wandering, for example during moments of internally focussed deliberate autobiographical planning^75^. The variations between the RDPs that we observed in this study could provide another view on this type of behavior.

The brain parcellation that is subsequently derived based on the RDP assignments is meaningful for various reasons. First, even if in a fully data-driven procedure, the majority of the voxels end up in labeled region with striking interhemispherical symmetry. Second, the consistency of our atlas parcels connectivity with previous literature confirms that the voxel grouping based on dFC remains coherent with previous stationary FC explorations, having the additional advantages of being whole-brain, without a-priori assumptions and based on FC temporal dynamics. In fact, only a few previous reports consider dFC to determine a parcellation^63,78^ and no previous work performs the dFC analysis at the voxel level. A recent effort to a multimodal parcellation demonstrated the advantages of integrating anatomical, functional, topographic and connectivity information to drive cortical parcellations^32^. Our findings suggest how the inclusion of connectivity dynamics could further improve the results, aiming to maximize parcellation accuracy and faithfulness to the actual aggregation of different brain compartments.

#### Precuneus

The precuneus is already known for its functional diversity; based on stationary FC, previous work has already highlighted its subdivisions^62,63^. The three subdivisions of the dorsal precuneus that we obtain resemble the ones described in a previous work^62^. When analyzing the dFC of the different portions, however, we can detect differences with their findings, based on stationary FC analysis and indicating selective connectivity of the anterior, central and posterior precuneus to sensorymotor, association and visual cortices, respectively. In our case, in fact, the three subportions of the precuneus result to be all connected, in terms of dFC, with anterior salience/SMA, visual and temporal areas, but with distinct selected subregions of these networks and in different combinations. A previous study^63^ used seed-based dFC to investigate the connectivity of the same three subregions and found, consistently with us, that these are not totally functionally segregated systems, but they are instead involved in common patterns of connectivity, temporally alternating.

Further, the dFC of the ventral precuneus, here alternately involving FPN, language network and different regions of the DMN, confirmed the heterogeneity of the latter, showed by many recent reports and previously discussed.

#### Cerebellum

Even though differences between functional and anatomical parcellations can be expected, we compared our cerebellar parcellation against anatomical atlases (AAL and SUIT). We did find a certain degree of similarity, but also a number of anatomical cerebellar regions that ended up with the same label in our atlas, indicating they exhibited similar FC dynamics. Also, some of our functional subdivisions do not correspond with anatomical boundaries, which might indicate a richer set of functionality for the same anatomical area.

We then compared against other functional parcellations based on stationary FC reports^79–83^ —so far, no other study considered FC dynamics to parcellate the cerebellum. In our dFC-driven parcellation, the ROIs include cerebellar portions that selectively connect to distinct large-scale functional networks. A resemblance with the functional parcellation in 17 regions obtained in a previous study^79^ can be found, even if in that case, the number of regions was chosen a-priori, based on the number of cortical networks explored, while the initial voxelwise dFC analysis has here the advantage of deriving the whole-brain parcellation (including the number of regions) from the data, based only on their connectivity properties.

More specifically, the cerebello-cortical long-range connectivity that we retrieved appears consistent with previous literature. First of all, we found the cerebellum to be functionally connected to many different regions of the cortex, apart from known neuronal fiber projections to the motor areas. This supports the theory which sees this complex structure involved in a wider range of functions than traditionally thought; i.e., including cognitive and emotional tasks, beside the executions and planning of movement^79–81,83,84^. In particular, Crus I and II, as well as lobule IX, were found to be connected to DMN and FPN cortical regions, in accordance with several previous analyses^79,80,82,83^. Further, lobules IV-V-VI were functionally related to sensorimotor^79,80,83^ and motor areas^82,83^, as well as salience network^80^.

Finally, controversial findings were reported in the past concerning the connections to primary visual and auditory areas. No cerebellar connection to these systems was reported in fact by ref.^79^, while^83^ defined a primary sensorimotor zone of the cerebellum connected to visual and auditory areas, besides motor and sensorimotor ones. Here, we found that cortical projections of the cerebellum to these regions are limited (see Fig. 5B, last panel on the right), as we can attribute that to only a cerebellar ROI including a very small portion of the Vermis VII-VIII. Therefore, in terms of our evidence based on dFC, the auditory and visual areas are not among the main cerebellar connections.

## Methods

### Subjects and Acquisitions

The study included *N*_*S*_ = 54 healthy subjects selected from the Human Connectome Project (HCP)^85^ 900 subjects release. All experiments were reviewed and approved by the local institutional ethical committee (Swiss Ethics Committee on research involving humans). Informed consent forms, including consent to share de-identified data, were collected for all subjects (within the HCP) and approved by the Washington University institutional review board. All methods were carried out in accordance with relevant guidelines and regulations. Subjects were selected from the HCP S900 database with the following criteria: age between 31 and 35 years old, cognitive status (MMSE) > 28, image reconstruction info = 3 T MR r227. The structural 3D MPRAGE T1-weighted sequence (TR = 2400 ms, TE = 2.14 ms, TI = 1000, flip angle = 8 deg, FOV = 224 × 224 mm, voxel size = 0.7 mm isotropic) and both sessions of the two resting-state gradient-echo EPI sequences (1200 frames, TR = 720 ms, TE = 3.31 ms, flip angle = 52 deg, FOV = 208 × 180 mm, voxel size = 2 mm isotropic, multiband factor = 8)^86–88^ were considered for the analysis.

### Preprocessing

We started from the minimally preprocessed datasets supplied from HCP, in which spatial distortions have been minimized and the data have been aligned across modalities and across subjects to Montreal Neurological Institute (MNI) standard space using appropriate volume-based and surface-based registration methods^89^. Additionally, spatial smoothing (Gaussian kernel with FWHM = 5 mm) was performed using SPM8 (FIL,UCL,UK). The first 10 volumes were discarded so that the fMRI signal achieves steady-state magnetization, resulting in *T* = 1190 time points considered. Voxelwise timecourses were then detrended (linear and quadratic trends) and nuisance variables were regressed out using the DPARSF toolbox^90^. These included six motion parameters and average white matter and cerebrospinal fluid signals, obtained from standard white matter and ventricular masks mapped to the subjects’ fMRI space and masked with individual segmentation maps^60^. The timecourses were then band-pass filtered in the range [0.0167–0.15 *Hz*] to enhance resting-state fluctuations. To restrict the analysis to voxels belonging to gray matter (GM), a standard GM parcellation in MNI coordinates (IIT_GM_Destrieux_mask, from Illinois Institute of Technology (IIT) Human Brain Atlas)^11^ was resliced to fMRI resolution and masked with the mean functional volume of the population. This was then used to mask individual fMRI images. In this way, common locations for every subject were considered (number of voxels *N*_*V*_ = 109′783). Finally, the global signal at every time point was removed to discard the global component from the dFC-driven clusters retrieved in the following.

### Sliding-Window Functional Connectivity at the Voxel Level

Let **X** ^(*s*)^ denote the *N*_*V*_ × *T* fMRI data matrix for subject *s*, *s* = 1, …, *N*_*S*_, containing in its rows the preprocessed timecourses of voxels located in GM. A sliding window approach with window length *N*_*T*_ = 83 TR (equivalent to 60 sec) and step Δ = 5 TR (3.6 sec) was adopted, leading to split the original matrix into *N*_*W*_ windows to be analyzed (Fig. 1A). For each windowed data matrix ${{\bf{X}}}_{i}^{(s)}$, *i* = 1, …, *N*_*W*_, after normalizing the timecourses using z-scoring and division by the square root of the window length, we applied the fast eigenvector centrality method, initially proposed by ref.^53^ for stationary FC. This algorithm approximates the connectivity matrix ${{\bf{C}}}_{i}^{(s)}$ of window position *i* by the outer product of the first eigenvector ${{\bf{u}}}_{i}^{(s)}$, which is optimal in terms of explained variance:

1 $${{\bf{C}}}_{i}^{(s)}={{\bf{X}}}_{i}^{(s)}{({{\bf{X}}}_{i}^{(s)})}^{T}\approx {\lambda }_{i}^{(s)}{{\bf{u}}}_{i}^{(s)}{({{\bf{u}}}_{i}^{(s)})}^{T},\,{\rm{where}}\,{{\bf{C}}}_{i}^{(s)}{{\bf{u}}}_{i}^{(s)}={\lambda }_{i}^{(s)}{{\bf{u}}}_{i}^{(s)}.$$

To compute the leading eigenvector ${{\bf{u}}}_{i}^{(s)}$, the numerical algorithm first computes ${({{\bf{X}}}_{i}^{(s)})}^{T}{{\bf{u}}}_{i}^{(s)}$ (*N*_*T*_ × 1), which is then premultiplied by **X** ^(*s*)^ to obtain the result of ${{\bf{C}}}_{i}^{(s)}{{\bf{u}}}_{i}^{(s)}$. Therefore, the matrix ${{\bf{C}}}_{i}^{(s)}$ does not need to be computed nor stored explicitly, as we will express any operations on ${{\bf{C}}}_{i}^{(s)}$ as equivalent operations using the implicit representation provided by ${{\bf{u}}}_{i}^{(s)}$. To ensure that only deviations of connectivity from each subject’s stationary FC are captured^60^, we need to center the dFC values for each connection; i.e., we want to subtract from each windowed connectivity matrix ${{\bf{C}}}_{i}^{(s)}$ the subject-wise stationary connectivity matrix **C** ^(*s*)^. To make this operation feasible, we first approximated **C** ^(*s*)^ by a rank-*M* approximation:

2 $${{\bf{C}}}^{(s)}={{\bf{X}}}^{(s)}{({{\bf{X}}}^{(s)})}^{T}\approx \sum _{k=1}^{M}\,{\mu }_{k}^{(s)}{{\bf{v}}}_{k}^{(s)}{({{\bf{v}}}_{k}^{(s)})}^{T},\,{\rm{where}}\,{{\bf{C}}}^{(s)}{{\bf{v}}}_{k}^{(s)}={\mu }_{k}^{(s)}{{\bf{v}}}^{(s)}.$$

The effect of centering ${{\bf{C}}}_{i}^{(s)}$ can then be computed as

3 $$({{\bf{C}}}_{i}^{(s)}-{{\bf{C}}}^{(s)}){{\bf{u}}}_{i}^{(s)}={{\bf{X}}}_{i}^{(s)}{({{\bf{X}}}_{i}^{(s)})}^{T}{{\bf{u}}}_{i}-\sum _{k=1}^{M}\,{\mu }_{k}{{\bf{v}}}_{k}^{(s)}{({{\bf{v}}}_{k}^{(s)})}^{T}\,{{\bf{u}}}_{i}^{(s)},$$

which can be incorporated in the eigenvector centrality computatation and where the products are implemented from right to left and only involve matrix-vector products. The computation of the *M* largest eigenvectors of **C** ^(*s*)^ is done using the ARPACK software library that is available in Matlab; we choose *M* = 50.

### Representative Dominant Patterns

The dominant patterns ${{\bf{u}}}_{i}^{(s)}$ were then concatenated across windows and subjects into a matrix **U** of size *N*_*V*_ × *N*_*W*_ *N*_*S*_ and *k*-means clustering based on cosine similarity was applied to obtain RDPs (Fig. 1B); i.e., the *K* patterns that could be consistently identified using 10-fold resampling. For a range of *K* = 1, …, 30, we divided the matrix of concatenated dominant patterns into 10 random partitions and left one out for testing. After performing clustering on the training set, we attributed the obtained clusters to the instances of the test set by minimizing the distance between test and cluster centroids. For each cluster, the average distance between the assigned instance and the cluster centroid was computed. The worst-case largest average distance was retained as the consensus measure to be minimized as a function of *K*. The centroids of the final clustering are the RDPs and denoted as **r**_*k*_, *k* = 1, …, *K*. It is important to notice that the sign of the RDPs is irrelevant, as inverting the sign leads to the same connectivity matrix; i.e., **uu** ^*T*^ = (−**u**) (−**u** ^*T*^).

The computation time was approximately 30 mins per subject for preprocessing, and 15 mins per subject for the estimation of dominant patterns. The estimation of RDPs by concatenating all subjects’ dominant patterns and performing clustering, took approximately 2 hours (timings obtained using our Matlab implementation on a server (Dual Intel Xeon Processor 2.5 GHz/128 GB RAM) running the Ubuntu 14.04 distribution).

### Parcellations Reflecting Long-Range and Fine-Scale Organization

Based on the RDPs **r**_*k*_, every voxel was assigned a label corresponding to its positive/negative contributions to each of the RDPs. Mathematically, the label for a voxel *n* was derived using the following binary code:

4 $${\rm{label}}(n)=\sum _{k=1}^{K}\,{2}^{k-1}\frac{{\rm{sign}}({{\bf{r}}}_{k}(n))+1}{2}.$$

Consequently, voxels that show similar behavior during different patterns of fluctuations of dFC will end up with the same label. These labels identify dFC patterns reflecting long-range interactions, since they are made out of clusters distributed across the brain. The degree of distribution of each pattern was computed in terms of average Euclidean distance between all pairs of non-contiguous regions belonging to the pattern itself. The symmetry of the dFC patterns was evaluated in terms of proportion of voxels on the two hemispheres and symmetry index, computed as: SI = (#*L* − #*R*)/((#*L* + #*R*)/2), where #*L* and #*R* are the number of voxels in the left and right hemispheres, respectively.

Each of the dFC patterns was further split into its contiguous regions. We removed regions that contained less than 20 voxels (0.16 cm^3^). To analyze specific cortical regions of interest in the obtained atlas, existent masks of the precuneus^9^ and cerebellum^64,91^ were used to mask the atlas and selectively show the regions of interest of these areas.

### Reproducibility Analysis

To evaluate the reproducibility of the results across different sessions, the same analysis was carried out on the two distinct acquisitions provided by the HCP within the same resting-state session of every individual. The two datasets characterized by phase encoding in opposite directions were randomly mixed. The obtained RDPs were compared in terms of spatial correlation, while the degree of overlap between the retrieved dFC-driven parcellations was evaluated in terms of Adjusted Mutual Information, Rand Index and Adjusted Rand Index.

Further, the effect of adopting shorter/longer window lengths was evaluated by repeating the analysis with *N*_*T*_ = 42 TR (30 sec) and *N*_*T*_ = 125 TR (90 sec), and the retrieved RDPs and parcellations were compared.

## Electronic supplementary material

Supplementary Information
