# Supplementary material for: Dynamics of functional connectivity at high spatial resolution reveal long-range interactions and fine-scale organization
Source: Sci Rep. 2017 Oct 6;7:12773. doi: 10.1038/s41598-017-12993-1 (PMC5630612; doi:10.1038/s41598-017-12993-1)
Supplement: Supplementary file 1 — Supplementary Information [file 41598_2017_12993_MOESM1_ESM.pdf]

Dynamics of functional connectivity at high spatial resolution reveal long-range interactions and fine-scale organization

Maria Giulia Preti[1,2,*] **and Dimitri Van De Ville**[1,2]

[1]Institute of Bioengineering, Center for Neuroprosthetics, Ecole Polytechnique Fédérale de Lausanne (EPFL), Lausanne, Switzerland
[2]Department of Radiology and Medical Informatics, University of Geneva, Geneva, Switzerland
[*]maria.preti@epfl.ch

SUPPLEMENTARY MATERIAL

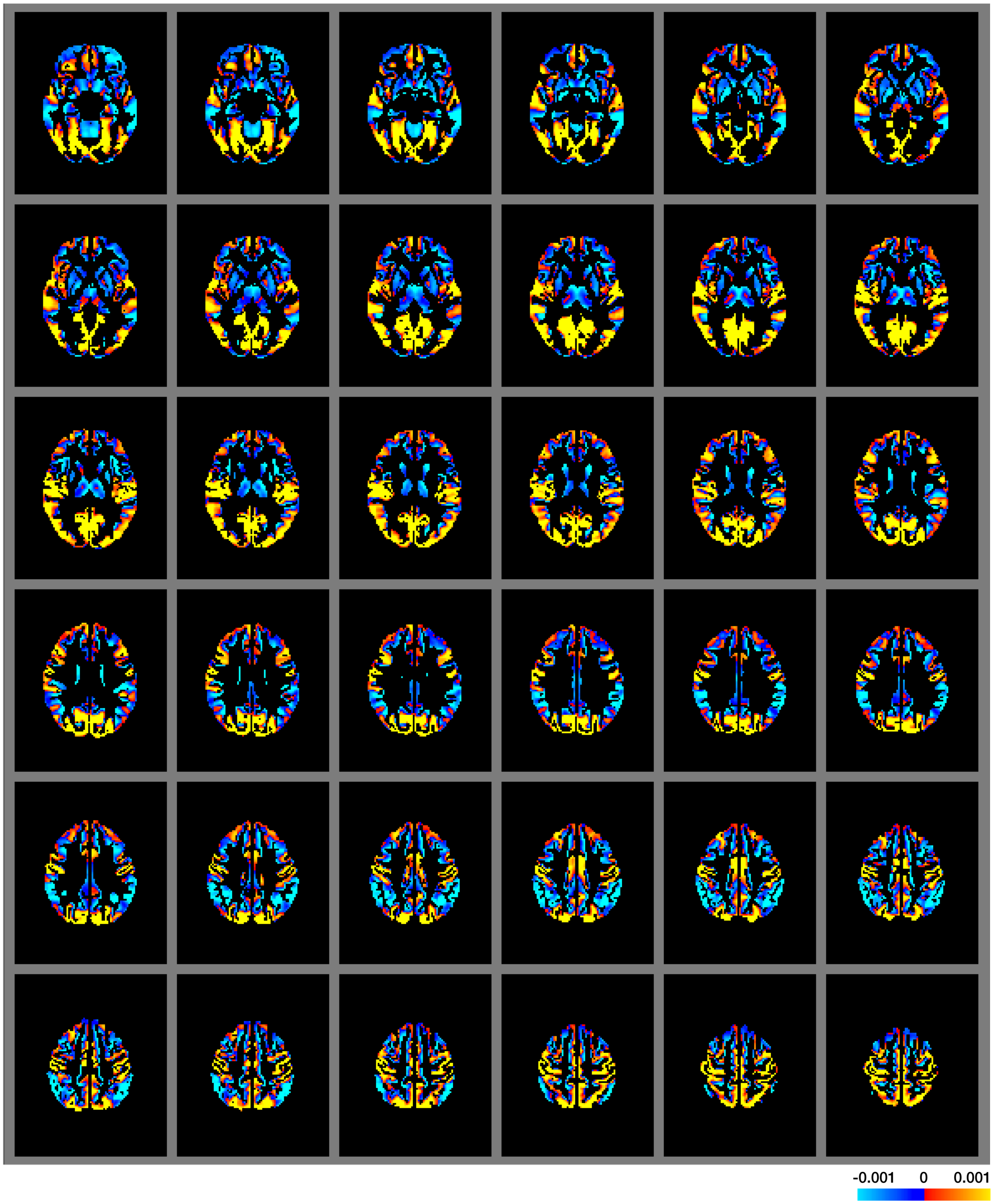

Figure S1. Multiple axial views of RDP1. MNI coordinates: from z=-12 (top left) to z=58 (bottom right).

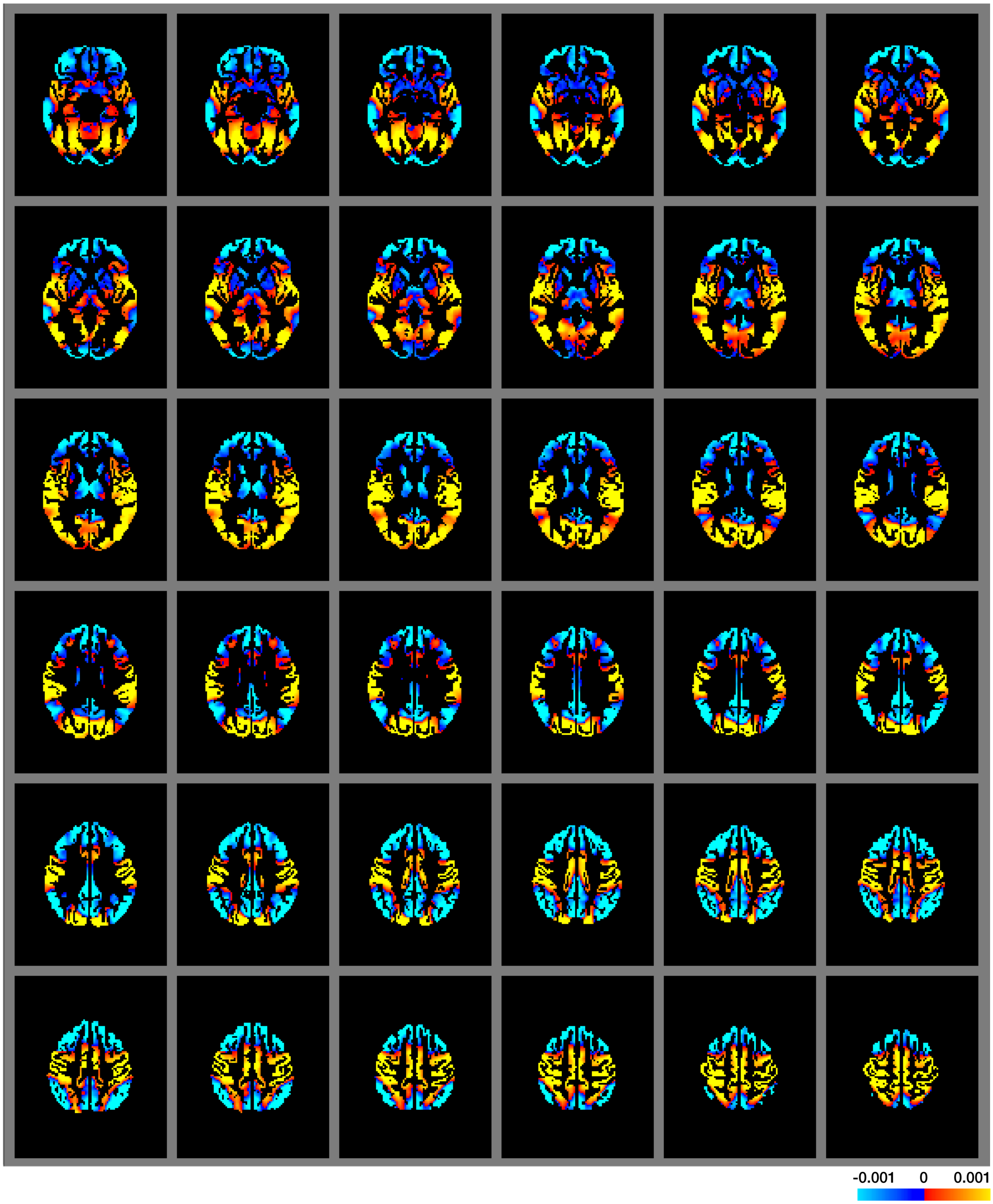

Figure S2. Multiple axial views of RDP2. MNI coordinates: from z=-12 (top left) to z=58 (bottom right).

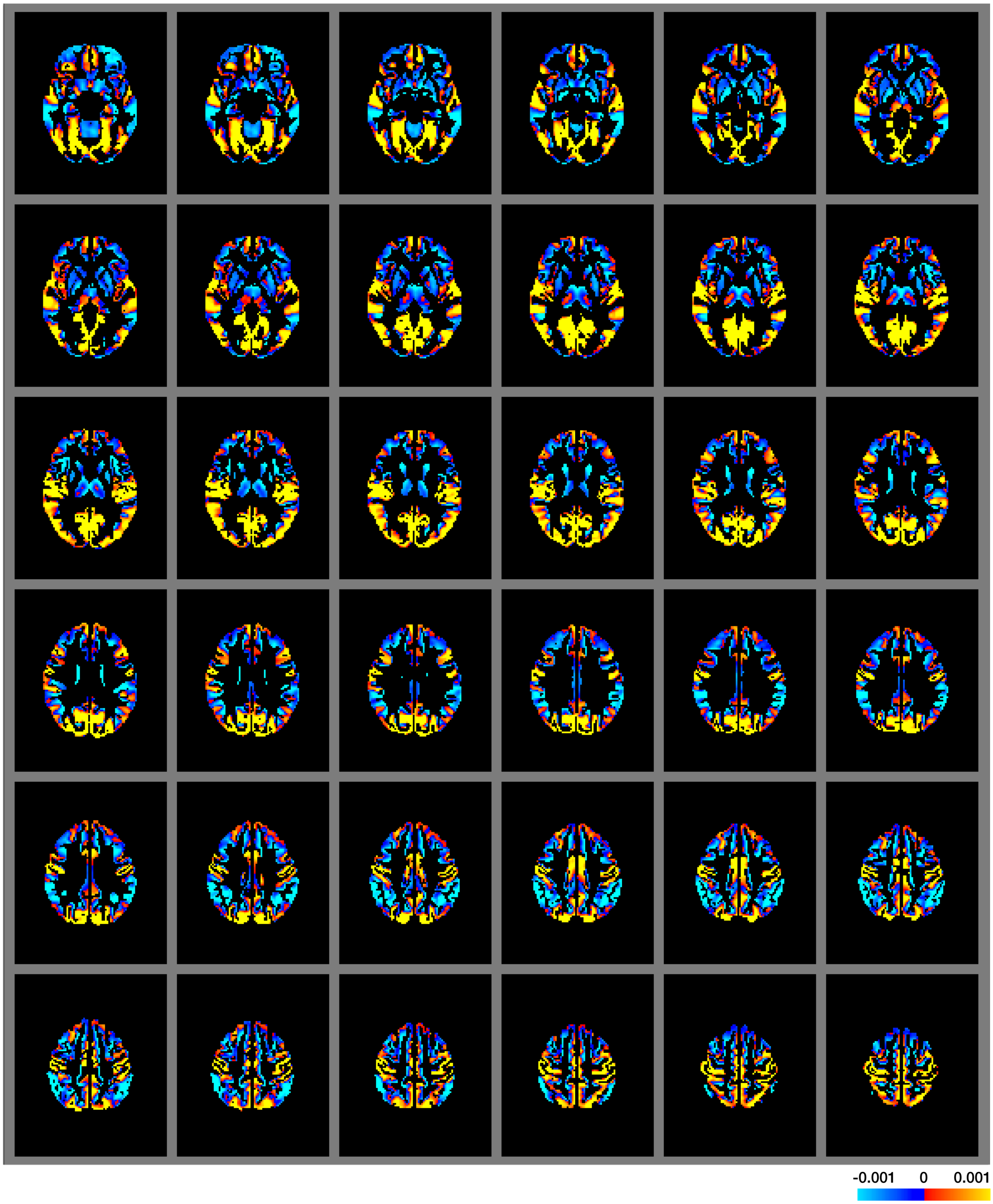

Figure S3. Multiple axial views of RDP3. MNI coordinates: from z=-12 (top left) to z=58 (bottom right).

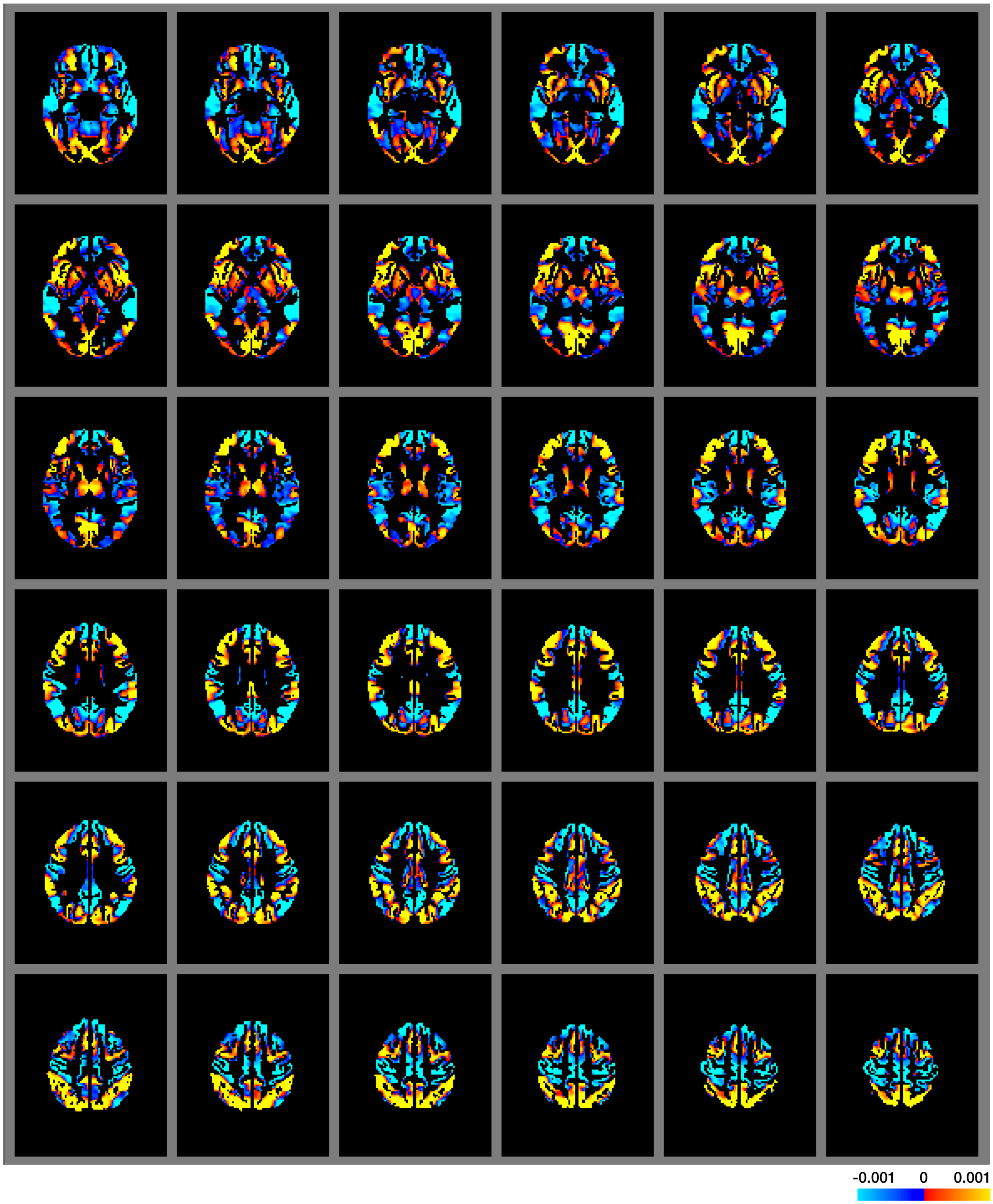

Figure S4. Multiple axial views of RDP4. MNI coordinates: from z=-12 (top left) to z=58 (bottom right).

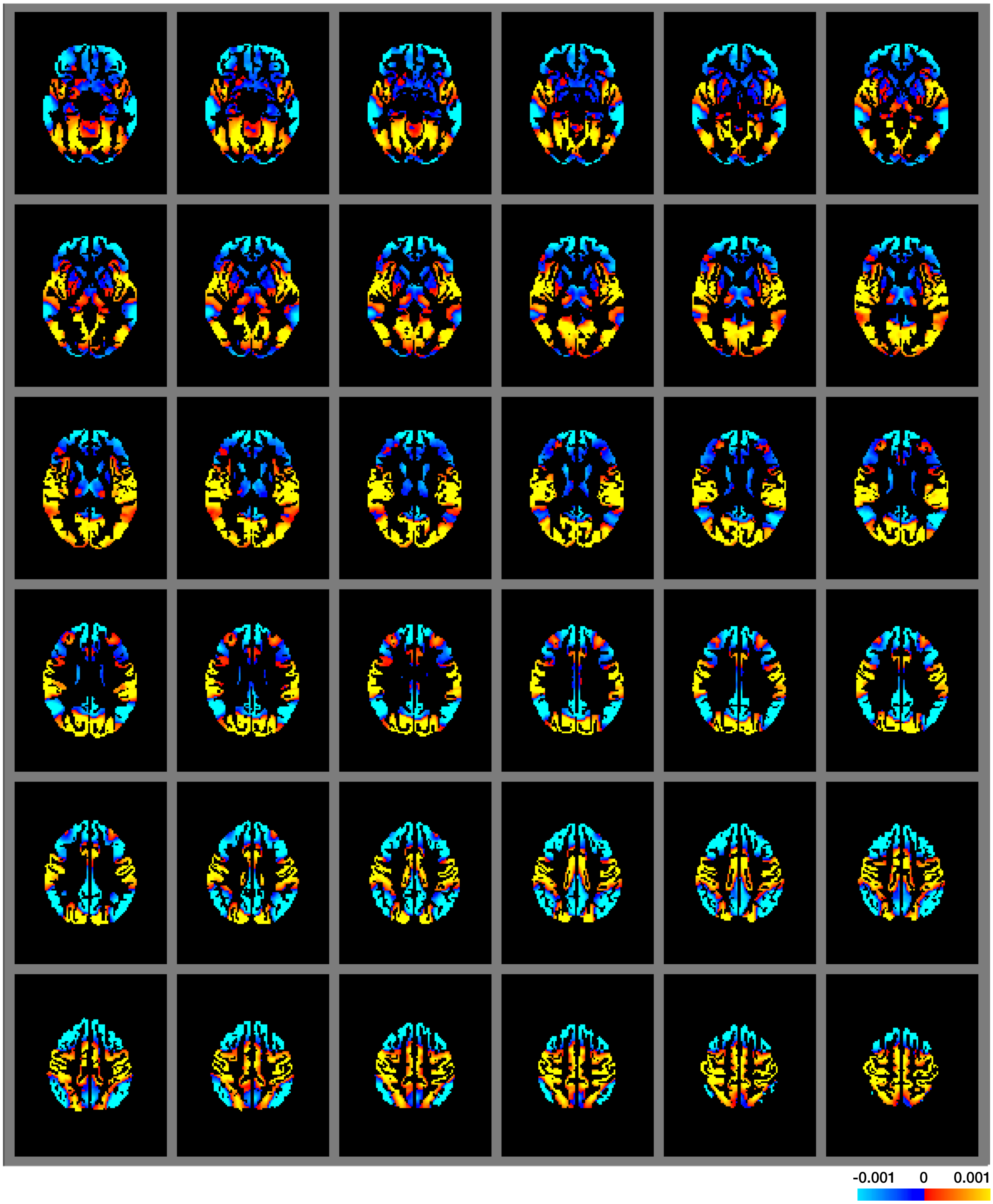

Figure S5. Multiple axial views of RDP5. MNI coordinates: from z=-12 (top left) to z=58 (bottom right).

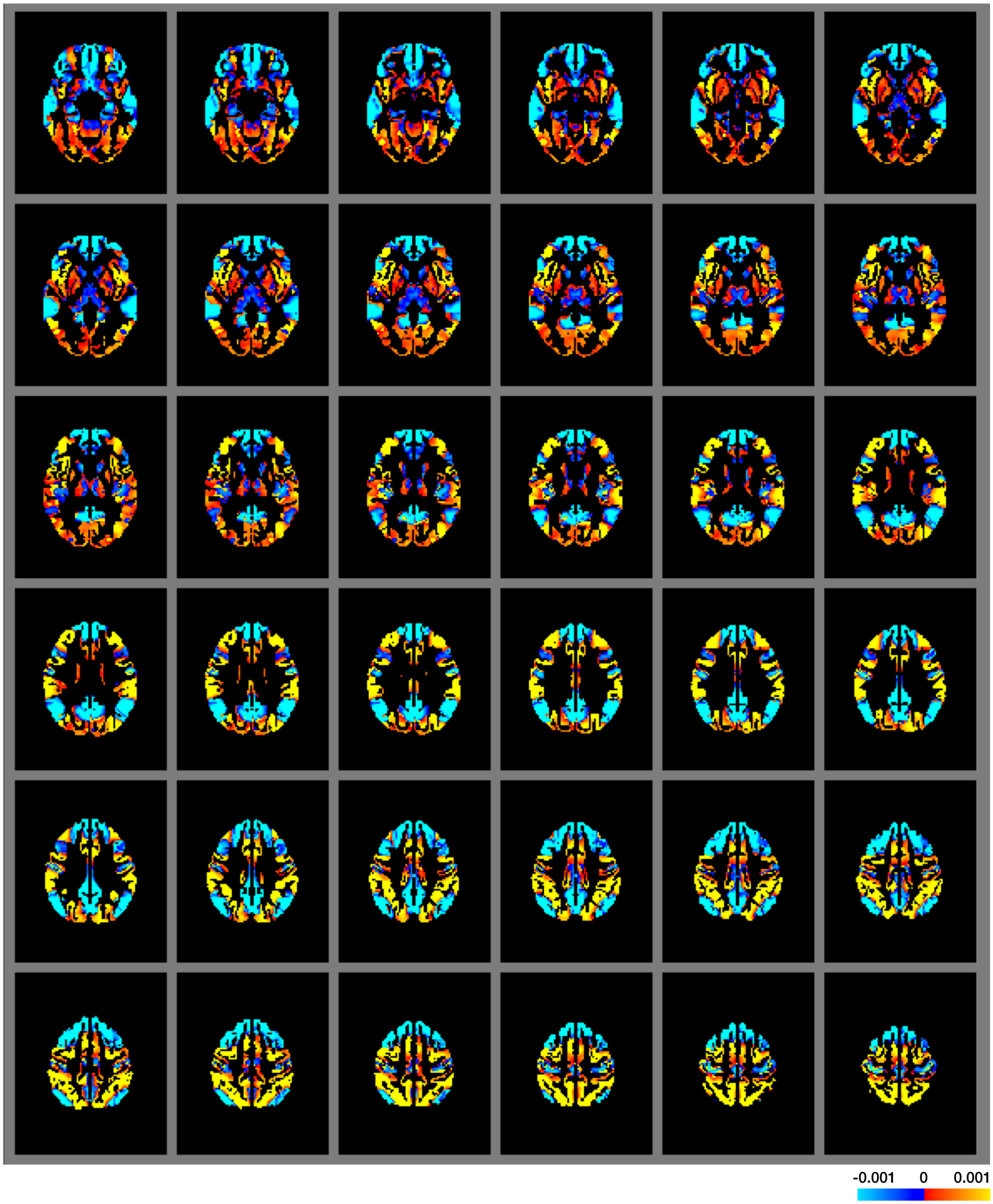

Figure S6. Multiple axial views of RDP6. MNI coordinates: from z=-12 (top left) to z=58 (bottom right).

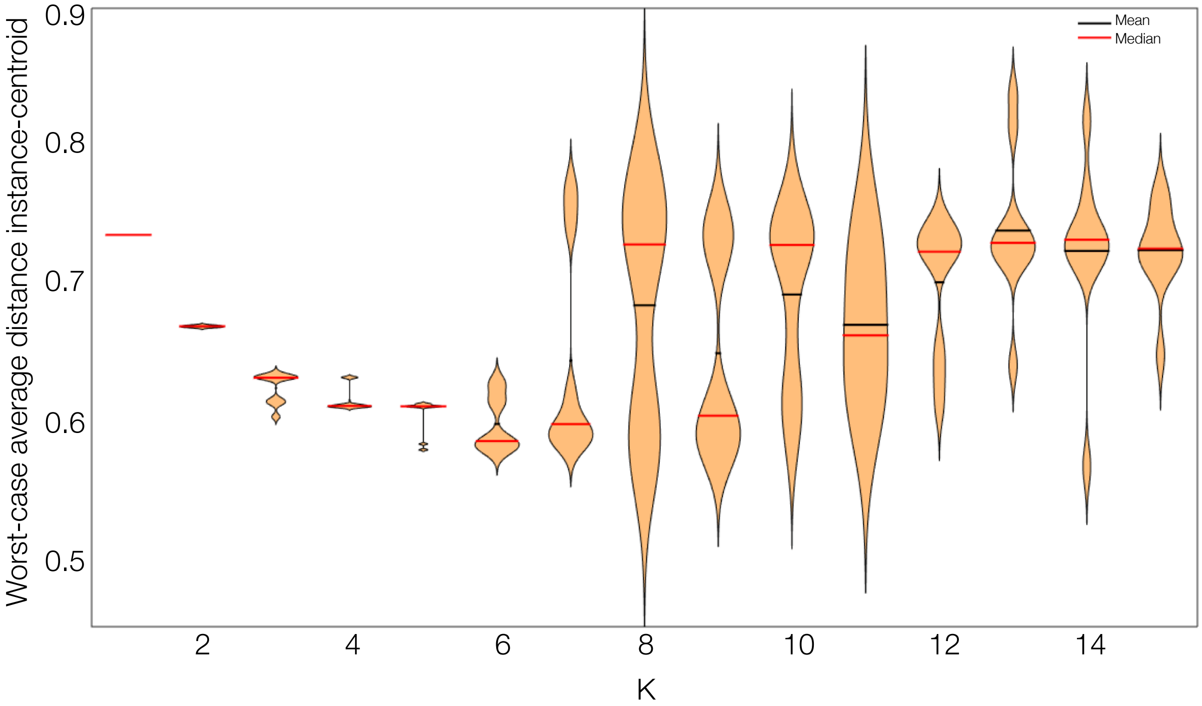

Figure S7. Violin plots illustrating the cross-validated clustering error for different K. The worst-case average distance between each instance and the centroid of the assigned cluster is shown in function of the number of clusters used K (for $K = 1, \ldots, 15$).

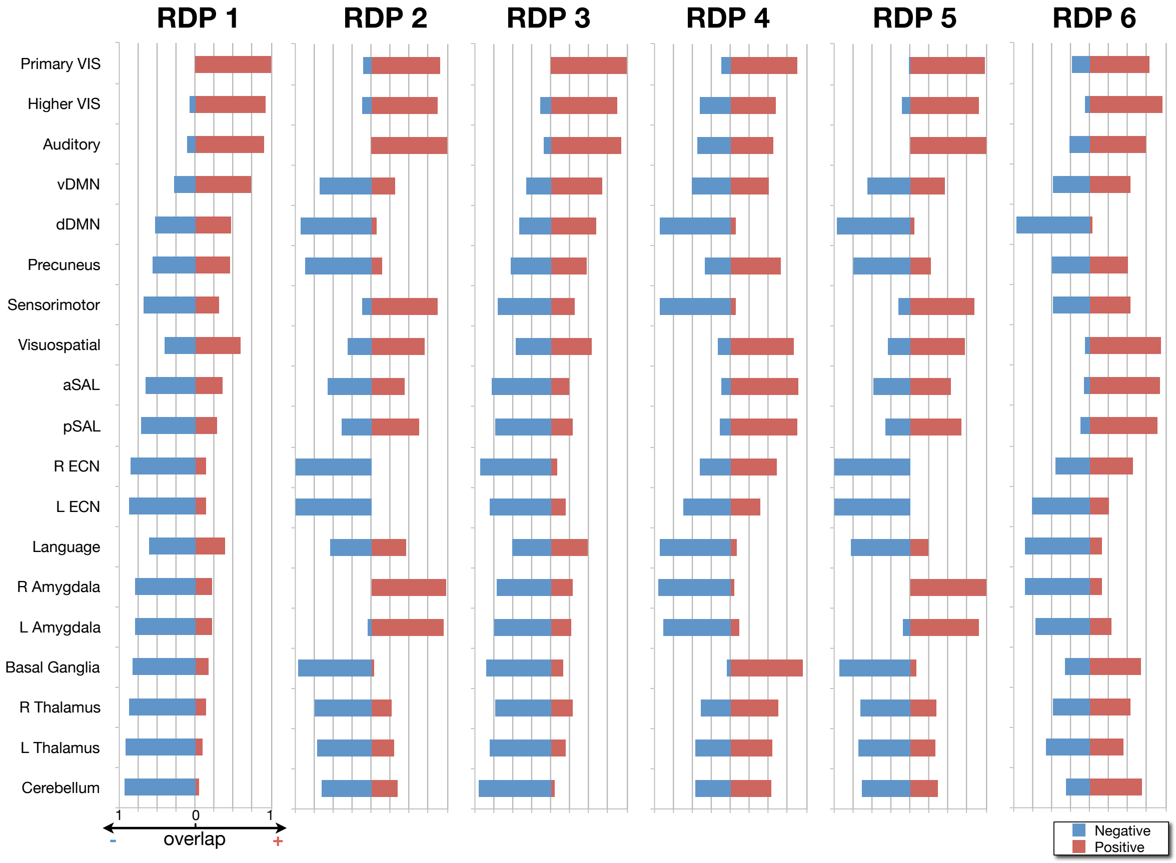

Figure S8. Percentages of overlap between each representative dominant pattern (RDP) (positive and negative parts) and the main resting-state networks (RSNs) and subcortical regions. The 14 RSNs found by Shirer et al. (2012) and selected subcortical AAL regions (thalamus, amygdala and cerebellum) are chosen as regions of interest (ROIs). The overlap is relative with respect to the ROI; therefore, since the RDP covers the whole brain, the bar length (sum of percentages with positive and negative overlap) is always 1 by construction. RDPs are constituted of different overlap profiles with conventional networks and main subcortical regions, which points to segregation and dynamic behavior. Abbreviations: VIS=visual, vDMN= ventral default mode network, dDMN= dorsal default mode network, aSAL=anterior salience, pSAL= posterior salience, ECN= executive control network, R=right, L=left.

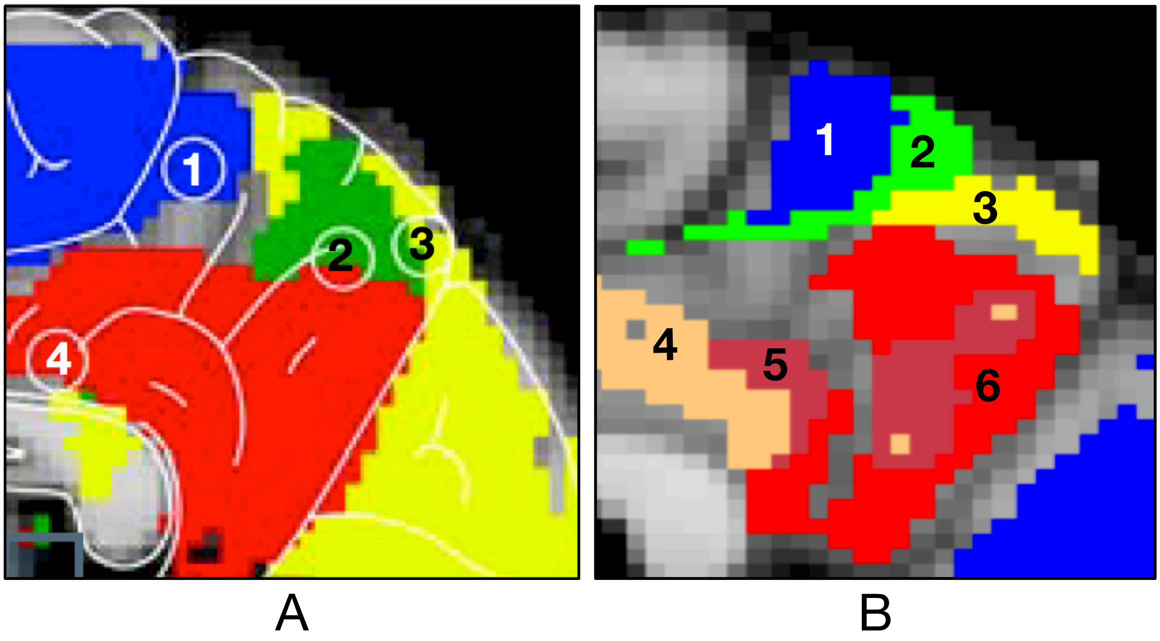

Figure S9. Precuneus subdivisions. (A) Seed-based FC parcellation as reported by Margulies et al. (2009, Fig. S7), using the four seeds indicated by the labels 1–4. (B) Our parcellation driven by dFC yields similar subdivisions of the precuneus dorsal part in (1) anterior, (2) middle and (3) posterior portions. However, in our case even the ventral portion of the posteromedial cortex, identified as one ROI in (A), is subdivided into 3 more regions (4, 5 6).

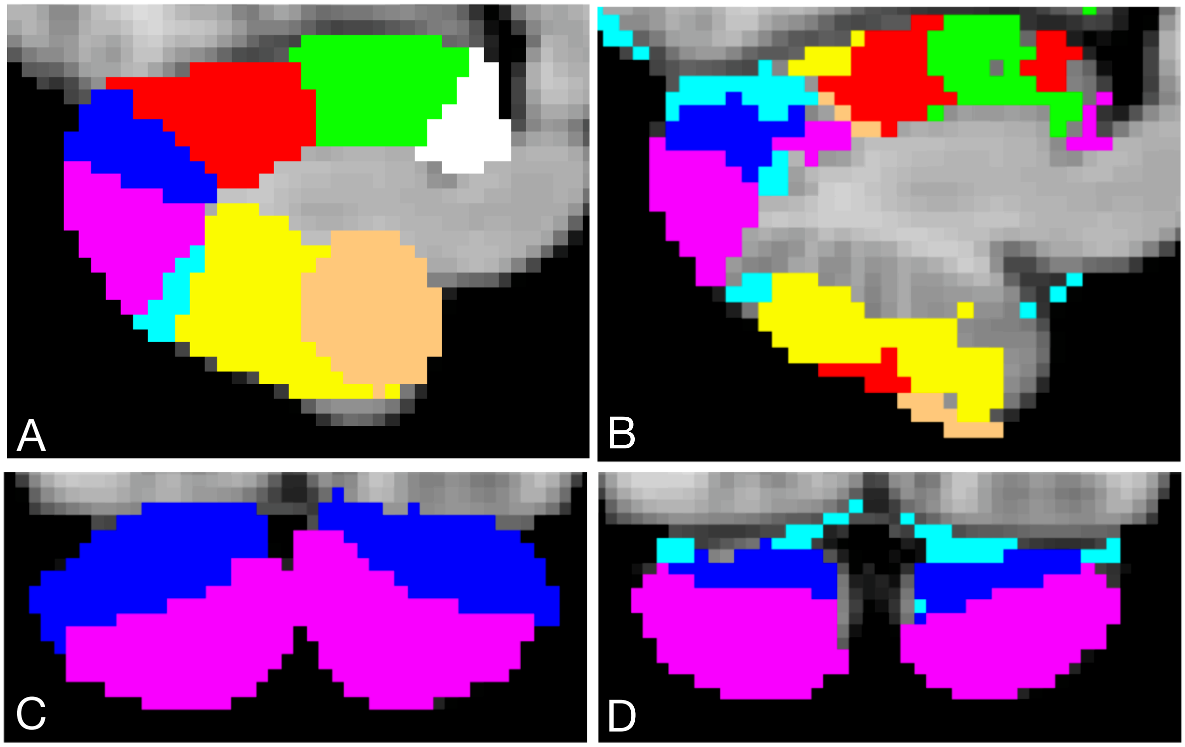

Figure S10. Cerebellar subdivisions in AAL atlas (A, C) and our parcellation (B, D). In the sagittal views (A, B), the partial consistency between the two parcellations can be appreciated, in the delineation of several subregions; e.g., lobules IV-V (green), VI (red), Crus I (blue) and Crus II (pink). Lobules VIII and IX (yellow and orange) result instead as one ROI in our atlas. In the coronal views (C, D), the division between Crus I and Crus II is shown.
